# Early cardiac events after haploidentical hematopoietic cell transplantation with post-transplant cyclophosphamide. subanalysis exploring cardiac toxicity conducted on behalf of GETH-TC

**DOI:** 10.3389/fimmu.2025.1571678

**Published:** 2025-05-01

**Authors:** Filipe R. Pinto, Enric Cascos, Estefanía Pérez-López, Mónica Baile-González, Carlos Martín Rodríguez, María Jesús Pascual Cascón, Marta Luque, Albert Esquirol, Carmen Martín Calvo, Felipe Peña-Muñóz, Inmaculada Heras Fernando, Itziar Oiartzabal Ormtegi, Adolfo Jesús Sáez Marín, Sara Fernández-Luis, Juan José Domínguez-García, Sara Villar Fernández, José Luis López Lorenzo, Miguel Fernández de Sanmamed Girón, Leslie González Pinedo, Lucía García, Ana Pilar González-Rodriguez, Tamara Torrado, Silvia Filaferro, Pascual Basalobre, Alberto López-García, Guillermo Ortí, Manuel Jurado Chacón, María Queralt Salas

**Affiliations:** ^1^ Clinical Hematology Department, Unidade Local de Saúde de Santo António – Hospital de Santo António, Oporto, Portugal; ^2^ Cardiology Department, Institut Clínic Cardiovascular (ICCV), Hospital Clinic de Barcelona, Barcelona, Spain; ^3^ Hematology Department, Complejo Asistencial Universitario de Salamanca/Instituto de Investigación Biomédica de Salamanca (IBSAL), Salamanca, Spain; ^4^ Hematology Department, Hospital Regional Universitario de Málaga, Málaga, Spain; ^5^ Hematology Department, Hospital de la Santa Creu i Sant Pau, Barcelona, Spain; ^6^ Hematology Department, Hospital Reina Sofía de Córdoba, Córdoba, Spain; ^7^ Hematology Department, Institut Català d’Oncologia - Hospital Duran i Reynals, Barcelona, Spain; ^8^ Hematology Department, Hospital General Universitario Morales Meseguer, Murcia, Spain; ^9^ Hematology Department, Hospital Universitario Donostia, Donostia, Spain; ^10^ Hematology Department, Hospital Universitario 12 de Octubre, Madrid, Spain; ^11^ Hematology Department, Hospital Universitario Marqués de Valdecilla (IDIVAL), Santander, Spain; ^12^ Hematology Department, Clínica Universidad de Navarra, Pamplona, Spain; ^13^ Hematology Department, Fundación Jiménez Díaz, Madrid, Spain; ^14^ Hematology Department, Hospital Universitario de Gran Canaria Doctor Negrín, Gran Canaria, Spain; ^15^ Hematology Department, Hospital Universitario Son Espases, Palma de Mallorca, Spain; ^16^ Hematology Department, Hospital Universitario Central de Asturias, Oviedo, Spain; ^17^ Hematology Department, Hospital Universitario de A Coruña, A Coruña, Spain; ^18^ Grupo Español de Trasplante de Progenitores Hematopoyéticos y Terapia Celular, Madrid, Spain; ^19^ Hematology Department, Hospital Universitari Vall d’Hebron, Barcelona, Spain; ^20^ Hematology Department, Hospital Universitario Virgen de las Nieves de Granada, Granada, Spain; ^21^ Hematopoietic Transplantation Unit, Hematology Department, Institute of Cancer and Hematological Diseases (ICAMS), Hospital Clínic de Barcelona, Barcelona, Spain

**Keywords:** Haplo-HCT, PTCY, early cardiac events, cardiovascular toxicity, AML, GVHD prophylaxis

## Abstract

**Introduction:**

Haploidentical allogeneic hematopoietic cell transplantation (haplo-HCT) using post-transplant cyclophosphamide (PTCY) has become a standard approach for patients lacking HLA-matched donors. While effective in reducing graft-versus-host disease (GVHD), concerns about PTCY-associated cardiovascular toxicity remain. This study investigates the incidence, predictors, and impact of early cardiac events (ECE) in haplo-HCT recipients.

**Methods:**

We conducted a retrospective, multicenter analysis of 268 patients with acute myeloid leukemia (AML) treated with anthracycline-based induction regimens and undergoing their first haplo-HCT with PTCY (50 mg/kg/day on days +3 and +4) between 2011 and 2022. ECEs, defined as any new cardiac event within 100 days post-transplant, were analyzed using cumulative incidence functions considering death and relapse as competing risks. Risk factors and the impact on non-relapse mortality (NRM) and overall survival (OS) were assessed via univariate and multivariate regression models.

**Results:**

The median patient age was 57 years (range: 18–79), and pre-transplant comorbidities included hypertension (22.4%), dyslipidemia (13.1%), diabetes mellitus (6.7%), and prior cardiac history (14.2%). ECEs occurred in 23 patients (8.6%) at a median of 19 days post-transplant (IQR: 5–66), with a day +100 cumulative incidence of 8.6% (95% CI: 6.1–12.3). The most frequent complications were pericardial effusion/pericarditis (43.5%), arrhythmias (30.4%), and heart failure (17.4%). Severe ECEs (CTCAE grade 3–4) were observed in 30.4% of cases, and four deaths (17.4%) were directly attributed to ECEs. Univariate analysis identified dyslipidemia (HR: 3.87, p=0.001), hypertension (HR: 2.76, p=0.015), and moderate-severe veno-occlusive disease (HR: 4.94, p=0.002) as significant predictors of ECE. ECEs were associated with lower OS (HR: 1.78, p=0.04) and higher NRM (HR: 2.87, p=0.005).

**Discussion:**

While the incidence of ECEs following haplo-HCT with PTCY was relatively low, their occurrence significantly worsened transplant outcomes. These findings underscore the importance of cardiovascular risk assessment and structured cardiac monitoring to mitigate complications in haplo-HCT recipients.

## Introduction

Haploidentical allogeneic hematopoietic cell transplantation (haplo-HCT) has become a viable option for patients lacking fully HLA-matched sibling or unrelated donors, with the use of post-transplant cyclophosphamide (PTCY)-based prophylaxis playing a pivotal role in its success. PTCY has proven highly effective in reducing both acute and chronic graft-versus-host disease (GVHD), facilitating the widespread adoption of haplo-HCT in clinical practice with favorable outcomes.

Despite these advantages, concerns about PTCY’s potential cardiovascular toxicity remain significant, since cyclophosphamide´s use has been linked to cardiac events (CE) in 1% to 17% of cases (1–4) when used as part of induction treatments, conditioning regimens and for GVHD prevention. Cardiovascular toxicity after allo-HCT is a well-recognized complication linked to increased risk for transplant-related mortality. It can manifest early after the stem cell infusion and during the late post-transplant follow-up. Recent studies conducted in patients receiving PTCY-based prophylaxis have reported incidences of early cardiac events (ECE) of 7.4% to 19%, with haplo-HCT patients appearing to be at an elevated risk (5–8). Hence, given the growing use of PTCY in haplo-HCT, understanding its cardiovascular implications is considered crucial.

Since 2024, the *Grupo Español de Trasplante y Terapia Celular* (GETH-TC) has undergone a retrospective and multicenter investigation with the objective of investigating the incidence and predictors of CE in allo-HCT performed with PTCY (9, 10). For this purpose, a homogeneous population of patients with acute myeloid leukemia (AML) treated with anthracycline-based induction therapies, also known to increase the risk for presenting cardiac toxicity have been selected (11, 12). The present complementary study specifically explores the incidence and predictors of ECE in haplo-HCT recipients receiving PTCY and investigates the impact of this complication on outcomes.

## Methods

### Study design and patient selection

This study is a retrospective, multicenter, registry-based analysis endorsed by the GETH-TC, a non-profit scientific society comprising members from all institutions performing HCT in Spain and Portugal, where sixteen transplant centers have participated.

Eligible criteria for patient selection were adult patients diagnosed with AML treated with at least one line of anthracycline-based induction therapies who underwent their first haplo-HCT with PTCY-based prophylaxis between 2021 and 2022. All patients received PTCY at a dose of 50mg/kg/day on days +3 and +4 and peripheral blood stem cell sources. Retrospective data collection was conducted via REDCap electronic data capture tools hosted by GETH-TC and finished on July 2024, The Ethics Committee of Hospital Clínic de Barcelona approved the study, which adhered to the ethical standards of the Declaration of Helsinki. No external funding was received.

This study represents a subgroup analysis of a previously published multicenter cohort. The same cohort was analyzed in two prior studies conducted by our group (9, 10), which examined early and late CE in broader allo-HCT populations. In contrast, the present study specifically focuses on ECE risk in haplo-HCT recipients, a subgroup with distinct immunological and cardiovascular challenges. This focus is particularly relevant given the heightened risk of cardiotoxicity in haplo-HCT, which may stem from increased cellular alloreactivity and the widespread use of PTCY as GVHD prophylaxis. These factors contribute to a unique inflammatory and endothelial injury profile that may predispose haplo-HCT recipients to early cardiac complications, warranting dedicated investigation.

### Early cardiac events

An early cardiac event (ECE) was defined as any new diagnosis of atrial or ventricular arrhythmias, heart failure (including a ≥10% reduction in left ventricular ejection fraction (LVEF) to <53%), myocardial infarction or ischemia, and pericardial complications (moderate/severe pericardial effusion or pericarditis) occurring within 100 days after HCT (5–7). Only the first ECE experienced by each patient was included in the analysis. Post-transplant hypertension (HTN) was not classified as a cardiac event. Cardiac complications were diagnosed according to standard clinical practices and the 2022 ESC Guidelines on Cardio-Oncology (13), and graded using the National Cancer Institute’s Common Terminology Criteria for Adverse Events (CTCAE), version 5.0.

### Pre-transplant cardiac function evaluation

Patients with pre-existing cardiac conditions included those with a history of arrhythmias, heart failure, ischemia, pericarditis, moderate/severe pericardial effusion, moderate/severe valvopathy, or other significant cardiac issues identified prior to haplo-HCT. Pre-transplant cardiac evaluations adhered to institutional guidelines, with all patients undergoing transthoracic echocardiography (ECHO) and electrocardiograms (ECG) before transplant. Those with significant abnormalities on ECHO or ECG were classified as having pre-existing cardiac disease, and patients with an LVEF below 45% were categorized as having pre-existing cardiac morbidity. Cardiac biomarkers were excluded from the study due to lack of standardization across centers.

### Haplo-HCT information and key definitions

Induction therapy, haplo-HCT eligibility, donor selection, conditioning regimens, and GVHD prophylaxis followed standard protocols at each participating center. Conditioning regimen intensity was tailored to each patient’s age and comorbidities. Acute and chronic GVHD (aGVHD and cGVHD) were graded according to established criteria (14–16). Complete remission (CR) and disease relapse were determined by the treating physician and recorded in the database.

### Statistical analysis

The primary outcome of interest was the diagnosis of an ECE after allo-HCT. The cumulative incidence function (CIF) of ECE was estimated using regression analysis, accounting for death and relapse as competing events. Cardiac events occurring after day +100 or following disease relapse were not accounted for. Thirty- and sixty-day mortality rates were calculated from the day of ECE diagnosis to death for patients who died during the subsequent follow-up.

Descriptive statistics were reported as counts and percentages, with Chi-square and Mann-Whitney U tests used to assess statistically significant differences across subgroups. Post-transplant follow-up of patients experiencing graft failure (GF) and undergoing second allo-HCT was censored at the time of the second infusion. Post-transplant outcomes were analyzed using Kaplan-Meier and cumulative incidence regression analyses, with GVHD analyses adjusting for death and relapse as competing events. Predictors of ECE, overall survival (OS), and non-relapse mortality (NRM) were evaluated using univariate and multivariate regression models (UVA and MVA). The impact of ECEs on OS and NRM was assessed using regression analysis across the entire patient cohort, treating ECE as time-dependent variable. All statistical tests were two-sided, with a significance level set at P<0.05. EZR software was used for all analyses (17).

## Results

### Baseline information and main haplo-HCT results

A total of 268 patients with AML were included. The baseline information is presented in **Table 1**. The median age was 57 years (range: 18–79), and 54.1% were male. Pre-transplant cardiac comorbidities included hypertension (HTN) in 60 (22.4%) patients, dyslipidemia in 35 (13.1%), diabetes mellitus (DM) in 18 (6.7%), and prior cardiac history in 38 (14.2%). The pre-transplant disease status showed that the majority of patients were transplanted in CR (n= 240; 89.6%), while 28 (10.4%) had active or refractory disease. Myeloablative conditioning (MAC) was used in 144 (53.7%) of patients, and 22 (8.2%) received total body irradiation (TBI), and all patients received peripheral blood grafts.

**Table 1:** Baseline characteristics of the study cohort and according to early cardiac toxicity.

Baseline Characteristics	All patients N= 268 (100%)	Patients with ECE N=23 (8.6%)	NO ECE N= 245 (91.4%)	P value
Age median (range) Older 59 years	56.9 (17.2 – 79.1) 117 (43.7)	60.8 (26.7 – 74.4) 13 (56.5)	56.6 (17.2 – 79.1) 104 (42.4)	0.122 0.193
Sex				
Male	145(54.1)	13 (56.5)	132 (53.9)	0.808
Female	123 (45.9)	10 (43.5)	113 (46.1)	
Relevant comorbidities				
Active smoker	32 (11.9)	1 (4.3)	31 (12.7)	0.330
Hypertension	60 (22.4)	10 (43.5)	50 (20.4)	0.011
Dyslipidemia	35 (13.1)	8 (34.8)	27 (11.0)	0.001
Diabetes Mellitus	18 (6.7)	2 (8.7)	16 (6.5)	0.696
Obesity (BMI>30)	22 (8.2)	1 (4.3)	21 (8.6)	0.704
History of Cardiac Disease	38 (14.2)	5 (21.7)	33 (13.5)	0.277
Prior treatment with cardiotoxic chemotherapy	8 (3.0)	1 (4.3)	7 (2.9)	0.517
Disease status prior allo-HCT				
Complete Remission	240 (89.6)	19 (82.6)	221 (90.2)	0.278
Refractory AML/Active Disease	28 (10.4)	4 (17.4)	24 (9.8)	
HCT-CI >3	37 (13.8)	6 (26.1)	31 (12.7)	0.096
Main Allo-HCT Information				
Intensity				
Myeloablative	144 (53.7)	9 (39.1)	135 (55.1)	0.142
Reduced intensity	124 (46.3)	14 (60.9)	110 (44.9)	
MAC Regimen containing Cy	32 (11.9)	2 (8.7)	30 (12.2)	1.01
Total Body Irradiation (TBI)	22 (8.2)	3 (13.0)	19 (7.8)	0.377
Median days neutrophil engraftment	18 (15 - 21)	18 (15 - 21)	18 (15 – 21)	0.811
Median days platelet engraftment	24 (16 - 31)	25 (16 – 36)	24 (16 – 30)	0.333
Graft failure	19 (7.1)	1 (4.3)	18 (7.3)	0.283
Vascular Post-transplant Endothelial Events				
Cumulative Incidence of VOD				
Day +100 all events	4.5 (2.4 - 7.4)	13.0 (3.1 - 30.2)	3.7 (1.8 - 6.6)	0.038
Day +100 moderate-severe	3.0 (1.4 - 5.6)	8.7 (1.4 - 24.6)	2.5 (1.0 - 5.0)	0.093
Cumulative Incidence of TMA				
Day +100 all events	4.9 (2.7 - 7.9)	4.3 (0.3 - 18.8)	4.9 (2.7 - 8.1)	0.889
Cumulative Incidence of GVHD				
Day +100 Grade 2–4 aGVHD	39.3 (33.4 – 45.2)	34.8 (16.1 – 54.3)	39.7 (33.5 – 45.9)	0.963
Day +100 Grade 3–4 aGVHD	10.6 (7.3 – 14.7)	13.0 (3.1 - 30.3)	10.4 (6.9 – 14.6)	0.184
2-year Moderate/Severe cGVHD	6.0 (3.3 - 9.7)	11.8 (1.7 - 32.4)	5.5 (2.9 - 9.3)	0.283
CMV reactivation	167 (62.3)	19 (82.6)	148 (60.4)	0.034
CMV disease	23 (8.6)	2 (8.7)	21 (8.6)	0.984
Relapse	64 (23.9)	6 (26.1)	58 (23.7)	0.804 0.210
Dead	107 (39.9)	12 (52.2)	95 (38.8)	
Directly secondary to ECE	4 (3.7)	4 (33.3)	0	
Bacterial Bloodstream Infections	107 (39.9)	9 (39.1)	98 (40)	0.970
Median Follow up	837 (5 – 3654)	219 (5 – 3285)	864 (235 – 3651)	0.030

Main Outcomes	All patients N= 268 (100%)	Patients with ECE N=23 (8.6%)	NO ECE N= 245 (91.4%)	P value
Overall Survival				
1-year	69.8 (63.8 – 74.9)	46.2 (25.1 – 65.0)	71.9 (65.8 – 77.1)	0.062
2-year		46.2 (25.1 – 65.0)	67.9 (61.6 – 73.4)	
Non-Relapse Mortality				
1-year	20.3 (15.7 – 25.3)	43.5 (22.6 – 62.7)	18.1 (1.36 – 23.2)	0.0117
2-year	20.3 (15.7 – 25.3)	43.5 (22.6 – 62.7)	18.1 (1.36 – 23.2)	
Cumulative Incidence of Relapse				
1-year	16.3 (12.1 – 21.0)	13.0 (3.1 – 30.3)	16.6 (12.2 – 21.5)	0.864
2-year	22.0 (17.1 – 27.2)	22.7 (7.8 – 42.3)	21.9 (16.9 – 27.4)	

aGVHD, acute Graft vs. Host Disease; allo-HCT, Allogeneic Hematopoietic Cell Transplantation; AML, Acute Myeloid Leukemia; BMI, Body Mass Index; cGVHD, chronic Graft vs Host Disease; Cy, Cyclophosphamide; CMV, Cytomegalovirus; ECE, Early Cardiac Events; HCT-CI, Hematopoietic Cell Transplantation-specific Comorbidity Index; MAC, Myeloablative Conditioning; TMA, Thrombotic Microangiopathy; VOD, Veno-occlusive Disease.

As reported in **Table 1**, neutrophil and platelet engraftments occurred at a median of 18 days (IQR: 15–21), and 24 days (IQR: 16–31), respectively, and GF was diagnosed in 19 (7.1%) patients. The day +100 CIF of veno-occlusive disease (VOD) and transplant-associated thrombotic microangiopathy (TA-TMA) were 4.5% and 4.9%. In addition, the CIF or grade 2–4 and grade 3–4 aGVHD at day +100 were 39.3% and 10.6%, respectively.

### Early cardiac events

**Figure 1:**
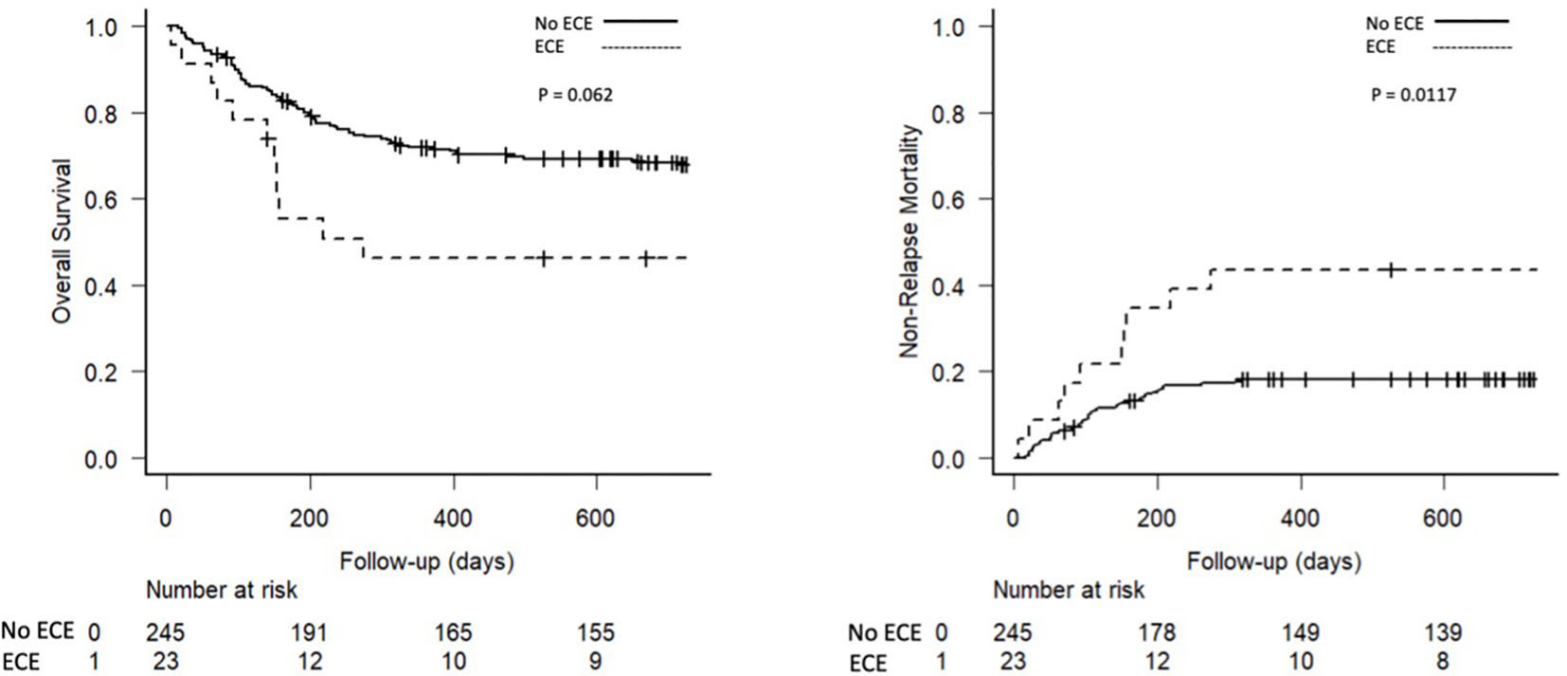
Risk factors for early cardiac events.

**Table 2** summarizes the key ECE data. ECEs occurred in 23 (8.6%) patients, after PTCY administration, at a median of 19 days after transplant (IQR: 5–66). As shown in **Figure 1**, theday +30 cumulative incidence of ECE was 5.2% (95% CI 3.0 – 8.4). Pericardial effusion or pericarditis were the most frequent complication (n=10, 43.5%), followed by arrhythmias (n=7, 30.4%) and heart failure (n=4, 17.4%), and lastly myocardial infarction or ischemia (n=2, 8.7%).

**Table 2:** Early cardiac events.

	N (%)	Grade of Severity (CTCAE)		Median days to Diagnosis (IQR)	Additional comment	Death secondary to CAE	Day + 30 Mortality Rate	Day + 60 Mortality Rate
All patients with ECE	23 (100)	1-2 3-4 5	13 (56.5) 6 (30.4) 4 (17.4)	19 (5-66)		4 (17.4)	3 (13.0)	5 (21.7)
Heart Failure	4 (17.4)	1-2 3-4 5	2 (50.0) 1 (25.0) 1 (25.0)	31 (7-66)	Two cases coursed with cardiac pulmonary edema	1 (25.0)	1 (25.0)	1 (25.0)
Myocardial infarction or ischemia	2 (8.7)	1-2 3-4 5	0 2 (100) 0	49 (3-49)	One case required percutaneal vascularization	0	0	0
Arrythmia	7 (30.4)	1-2 3-4 5	4 (57.1) 1 (14.3) 2 (28.6)	16 (10-28)	All supraventricular	2 (28.6)	1 (14.3)	2 (28.6)
Pericardiac effusion or pericarditis	10 (43.5)	1-2 3-4 5	7 (70.0) 2 (20.0) 1 (10.0)	26 (4-90)	7 cases of pericarditis and 3 of mod/sev pericardial effusions. No patient required pericardial drain.	1 (10.0)	2 (20.0)	2 (20.0)

CAE, Cardiac Adverse Events; CTCAE, National Cancer Institute’s Common Terminology Criteria for Adverse Events; ECE, Early Cardiac Events.

Severe ECEs (CTCAE grade 3–4) were diagnosed in 6 (30.4%) patients, with two cases manifesting with myocardial infarction, two with pericardial disorders, one with heart failure and another one with supraventricular arrhythmia. In addition, 4 (17.4%) patients died directly due to the cardiac complication (CTCAE grade 5), one due to supraventricular arrythmia, one due to heart failure, and two secondaries to pericardial diseases. Overall, mortality rates at 30 and 60 days after ECE diagnosis were 13% and 21.7%, respectively.

### Baseline information and post-transplant events of patients with ECE

The baseline information of patients with and without ECE were compared in between and described in **Table 1**. Patients with ECE tended to be older than those without ECE (median age 61 vs. 57 years, P=0.122). Pre-transplant cardiovascular risk factors HTN (43.5% vs. 20.4%, P=0.011) and DLP (34.8% vs. 11.0%, P<0.001) were more prevalent in the ECE group, but the proportion of patients with prior cardiac morbidity did not differ between groups (21.7% vs. 13.5%, P=0.277). Although the proportion of patients with refractory AML was more prevalent in patients with ECE (17.4% vs. 9.8%, P=0.278), the differences were not statistically significant. In addition, conditioning intensity, and TBI administration showed no differences between groups. As summarized in **Table 1**, neutrophil and platelet engraftment occurred with comparable timing in ECE and non-ECE groups (p = 0.811 and p = 0.333, respectively), while graft failure occurred less frequently in the ECE group (4.3% vs. 7.3%, P = 0.283). Rates of post-transplant vascular endothelial complications were compared between groups, revealing that patients with early cardiac events (ECE) had higher cumulative incidences of veno-occlusive disease (VOD) than those without ECE (Day +100: 13.0% vs. 3.7%, P=0.038). Specifically, three cases of VOD were identified among the 23 patients who experienced ECE, with a median onset of 12 days post-transplant (range: 11–62 days). The corresponding CE occurred on days 93, 49, and 13, respectively, and in two of these cases, VOD preceded the ECE. In contrast, the cumulative incidences of transplant-associated thrombotic microangiopathy (Day +100: 4.3% vs. 4.9%, P=0.889) and grades 2-4 (Day +100: 34.8% vs. 39.7%, P=0.184) and 3–4 aGVHD (Day +100: 13.0% vs. 10.4%, P=0.963) were similar between groups.

Additionally, CMV reactivation occurred significantly more frequently in patients without ECE than in those who developed ECE (82.6% vs. 60.4%, P = 0.034), although the clinical significance of this finding remains uncertain.

### Risk factors for ECEs

Predictors for ECE were investigated using UVA and MVA regression analyses. As described in **Table 3**, the UVA identified dyslipidemia (HR 3.87, 95% CI: 1.65–9.07, p=0.001), hypertension (HR 2.76, 95% CI: 1.22–6.24, p=0.015), and the diagnosis of moderate-to-severe VOD (VOD; HR 4.94, 95% CI: 1.27–19.22, p=0.002) as significant risk factors for ECE. In contrast, neither grade 2–4 nor grade 3–4 aGVHD showed a statistically significant association with ECE incidence (HR 1.98, P=0.178 and 2.24, P=0.251, respectively).

**Table 3:** Risk factors for early cardiac events.

Univariate Regression Analysis	Risk for Early Cardiac Events HR (95% CI)	P value
Age		
Continuous	1.02 (0.99 - 1.06)	0.15
Older than 60 years (vs. younger)	1.73 (0.76 - 3.93)	0.19
Sex		
Female (vs. Male)	0.90 (0.39 - 2.05)	0.8
Hypertension		
Yes (vs. no)	2.76 (1.22 - 6.24)	0.015
Dyslipidemia		
Yes (vs. no)	3.87 (1.65 - 9.07)	0.001
Diabetes Mellitus		
Yes (vs. no)	1.33 (0.315 - 5.69)	0.69
Obesity		
Yes (vs. no)	0.49 (0.07 - 3.54)	0.48
Previous cardiac pathology		
Yes (vs. no)	1.78 (0.656 - 4.83)	0.26
Disease Status Prior to Allo-HCT		
Active disease (vs. Complete Remission)	1.93 (0.65 - 5.73)	0.24
Conditioning Regimen		
MAC vs RIC	1.83 (0.80 - 4.23)	0.15
TBI		
Yes (vs. no)	1.70 (0.52 – 5.61)	0.38
Grade 2–4 aGVHD		
Time-dependent variable	1.98 (0.73 - 5.34)	0.178
Grade 3–4 aGVHD		
Time-dependent variable	2.24 (0.57 - 8.89)	0.250
VOD Yes (vs. no)	3.03 (0.95 - 9.67)	0.061
Time-dependent Mod-Sev	4.94 (1.27 - 19.22)	0.021

Multivariate Regression Analysis	Risk for Early Cardiac Events HR (95% CI)	P value
Hypertension		
(Yes vs. No)	2.08 (0.85 – 5.11)	0.109
Dyslipidemia		
(Yes vs. No)	2.59 (0.94 – 7.14)	0.065
Previous cardiac pathology		
(Yes vs. No)	1.14 (0.36 – 3.56)	0.825
Grade 3–4 aGVHD		
Time-dependent variable	1.96 (0.52 -7.36)	0.317
Moderate to Severe VOD		
Time-dependent variable	4.14 (1.29 – 13.2)	0.016

Allo-HCT, Allogeneic Hematopoietic Cell Transplantation; aGVHD, acute Graft vs. Host Disease; MAC, Myeloablative conditioning; RIC, Reduced intensity conditioning; VOD, Veno-occlusive disease.

TBI, Total body irradiation.

Variables included in the multivariate model were selected based on results reported in the univariate model and if considered clinically relevant for cardiac toxicity according to previous related publications. Post-transplant follow-up has been censored at 100 days. No event diagnosed after day +100 has been accounted for this analysis.

MVA confirmed an independent association between moderate-to-severe VOD and the risk of ECE (HR: 4.14, 95% CI: 1.29–13.2, *p*=0.016). In contrast, although hypertension (HR: 2.08, 95% CI: 0.85–5.11, *p*=0.109) and dyslipidemia (HR: 2.59, 95% CI: 0.94–7.14, *p*=0.065) showed a trend toward increased risk, their associations with ECE did not reach statistical significance.

### Transplant outcomes of patients with and without ECE

**Figure 2:**
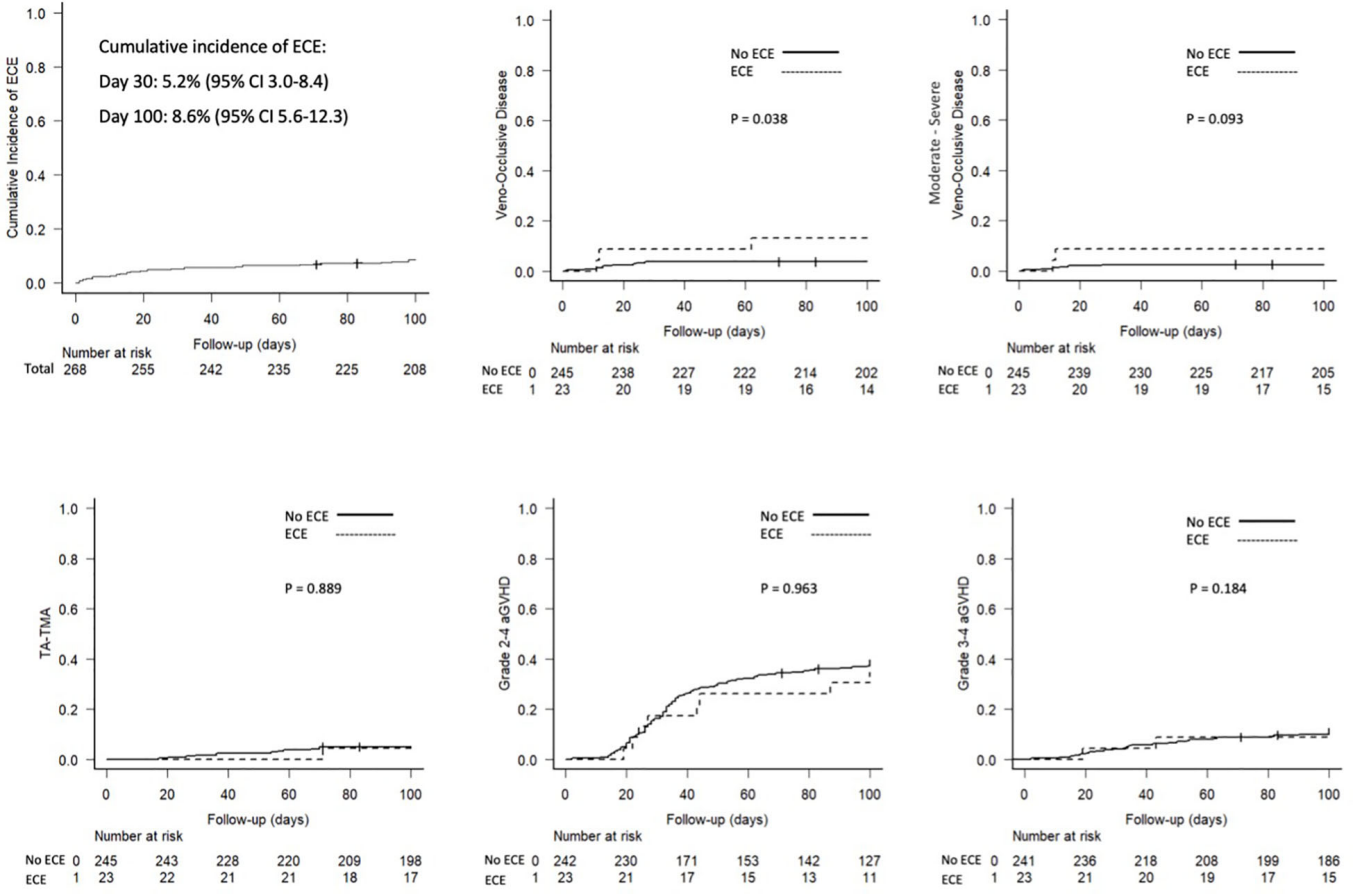
Main outcomes.

Overall, with a median follow-up of 27.9 months, 64 patients relapsed and 107 died. As reported in **Table 2**, the OS and NRM for all patients were 69.8% (95% CI: 63.8–74.9) and 20.3% (95% CI: 15.7–25.3). As reported in **Table 1** and **Figure 2**, higher NRM was documented in patients with ECE than in those who did not present cardiac complications during the first 100 days after haplo-HCT (1-year NRM of 43.5% vs. 20.3%, P=0.0117). Furthermore, a non-significant trend to shorter OS (1-year OS 46.2% vs. 71.9%, P=0.061) was observed in patients with ECE with comparable relapse rates (1-year cumulative incidence of relapse 13.0% vs. 16.6, P=0.864).

The effect of ECE on transplant outcomes was assessed using UVA and MVA for OS and NRM showing that ECEs had a significant negative impact on survival outcomes. As described in **Table 4**, in UVA, ECEs significantly increased the risk of mortality (HR 2.01, P=0.010) secondary to its impact on NRM (HR 2.12, P=0.008). The results of the UVA were confirmed in MVA showing that ECEs was an independent predictor of worse NRM (HR 2.19, 95% CI 1.01 – 4.76, p=0.048). Moreover, a trend toward reduced survival was observed in patients diagnosed with ECE than in the rest (HR 1.64, 95% CI 0.94 – 2.85, p=0.081).

**Table 4:** Impact of cardiac complications on transplant outcomes.

Univariate Analysis	OS HR (95% CI)	P value	NRM HR (95% CI)	P value
Cardiac Events				
All events (time dependent)	2.01 (1.18 – 3.44)	0.01035	2.12 (1.22 - 3.68)	0.007684

Multivariate Analysis	OS HR (95% CI)	P value	NRM HR (95% CI)	P value
Cardiac Events				
All events (time dependent)	1.64 (0.94 – 2.85)	0.081	2.19 (1.01 – 4.76)	0.048370
Patient Age				
Older than 59 (vs. younger)	1.24 (0.77 – 1.99)	0.379400	1.16 (0.60 – 2.24)	0.650500
HCT-CI				
>3 (vs. 0-3)	1.84 (1.14 – 2.99)	0.01295	2.60 (1.43 – 4.71)	0.001688
Disease status prior allo-HCT				
Refractory AML/Active Disease (vs. CR)	2.89 (1.76 – 4.77)	0.0000295		
Intensity				
RIC (vs. MAC)	1.18 (0.77 – 1.99)	0.492000	1.35 (0.70 – 2.61)	0.368200

Allo-HCT, Allogeneic Hematopoietic Cell Transplantation; AML, Acute Myeloid Leukemia; CR, Complete remission; HCT-CI, Hematopoietic Cell Transplantation-specific Comorbidity Index; HR, Hazard Ratio; MAC, Myeloablative conditioning; NRM, Non-relapse Mortality; OS, Overall Survival; RIC, Reduced intensity conditioning.

TBI, Total body irradiation.

The impact of cardiac events in outcomes has been investigated in the entire cohort of patients included in the study. CE variable has been treated as time dependent variable. Variables included in the multivariate model were selected based if considered clinically relevant for the transplant success. The variable HCT-CI has been included instead of the variable’s prior cardiac toxicity, obesity or mellitus diabetes.

Other factors associated with worse outcomes in MVA included a higher HCT-CI score than 3 and refractory AML or active disease at transplant. HCT-CI >3 was associated with worse OS (HR: 1.84, 95% CI 1.14 – 2.99, p=0.013) and NRM (HR: 2.60, 95% CI 1.43 – 4.71, p=0.002). Patients with refractory AML or active disease had a nearly threefold increased risk of death (HR: 2.89, 95% CI 1.76 – 4.77, p<0.001) and higher NRM (HR: 3.05, p<0.001).

## Discussion

This study evaluates the incidence, clinical manifestations, and predictors of ECE following haplo-HCT with PTCY. Our findings suggest that while the overall incidence of ECE is relatively low (8.6%), these events significantly impact NRM, supporting the importance of cardiac monitoring and prevention strategies in this setting.

Although the use of PTCY-based prophylaxis in haplo-HCT is now recognized as the standard of care, significantly improving outcomes by effectively controlling both acute and chronic GVHD, limited studies have investigated the incidence and predictors of early cardiac toxicity specifically in this transplant setting (5, 7, 8, 18, 19). The incidence of ECE (8.6%) observed in our study aligns with findings from prior research conducted in cohorts of allo-HCT that include patients undergoing haplo-HCT (5, 7, 8). Specifically, Duléry et al. reported a 19% incidence of early cardiotoxicity following haplo-HCT (5). Similarly, Lin et al. described a 21.9% incidence of ECE in a cohort of 176 patients where 80% of them (n=149) underwent haplo-HCT with PTCY-based prophylaxis (8). Notably, the present study utilizes the same multicenter cohort that was previously examined in two studies conducted by the GETH-TC (9, 10). However, while the previous studies investigated cardiac events in broader allo-HCT populations, the present study specifically focuses on ECE in haplo-HCT recipients, a subgroup with unique immunological and cardiovascular challenges.

Specifically, the first study conducted by our group identified an overall CE incidence of 9.2%, with ECE occurring in 4.8% of patients, including those receiving both PTCY-based prophylaxis and other GVHD prophylaxis regimens that did not contain PTCY. This study included 1,020 AML patients and principally confirmed the association between PTCY use and an increased risk of ECE in allo-HCT recipients. Consecutively, a subsequent analysis was conducted focused on CE after allo-HCT in the 453 AML patients who underwent allo-HCT with PTCY and different donor types. Although no significant differences in ECE incidences were observed according to donor type, rates of cardiac events occurring during the first 100 days after allo-HCT were higher in the haplo-HCT cohort than in the rest. This finding set the stage for the current study, which specifically looks at ECE in haplo-HCT recipients, emphasizing the need for a focused investigation into heart-related issues in this unique group of patients.

Consistent with prior studies, the clinical presentations of ECE following haplo-HCT were varied, with pericardial effusion/pericarditis (43.5%) and arrhythmias (30.4%) being the most frequently observed. Cardiac toxicity induced by Cy, predominantly studied in the context of conditioning regimens, involves mechanisms such as cardiomyocyte apoptosis, endothelial dysfunction, calcium imbalance, and injury to the endoplasmic reticulum and mitochondria (20, 21) These mechanisms contribute to a highly heterogeneous spectrum of clinical manifestations, including electrocardiographic abnormalities, pulmonary vascular congestion, pleural and pericardial effusions, and heart failure with preserved or reduced LVEF (4), posing significant challenges for the implementation of predictive or early detection strategies in this context.

Interestingly, higher incidences of ECE have been observed following haplo-HCT with PTCY compared to allo-HCT from HLA-matched donors (5, 7, 10). This difference has been attributed to increased T-cell alloreactivity and endothelial activation and dysfunction triggered by the less compatible stem cell products from haploidentical donors. Notably, endothelial activation has been linked to cardiovascular events in the general population, as well as in patients with predisposing conditions such as diabetes or atherosclerosis (22–24). These insights suggest that haplo-HCT patients may have an elevated baseline risk for endothelial injury, which could serve as a precursor for downstream cardiovascular complications, including ECE.

Considering these results, we examined whether post-transplant endothelial vascular complications such as VOD, aGVHD, or TMA contributed to ECE risk in our study cohort. Interestingly, the diagnosis of VOD emerged as a significant predictor for ECE. These findings underscoring a temporal and mechanistic link between VOD and CE in this population. VOD is characterized by substantial endothelial stress and inflammation, which may contribute to cardiac complications by inducing vascular congestion, amplifying microvascular injury, and promoting a pro-inflammatory and pro-thrombotic state (25). Additionally, results suggest that the endothelial damage occurring in VOD could act as a catalyst for acute cardiac events, particularly in the vulnerable early post-transplant period when endothelial repair mechanisms may still be impaired, and highlight the potential role of endothelial dysfunction in the pathogenesis of cardiac complications. Based on these results, studies exploring the association between endothelial injury and cardiovascular complications would be of interest to better understand the physiopathology of ECE in haplo-HCT settings.

In contrast, neither TMA nor aGVHD showed a correlation with higher incidence of cardiac complications. However, ECE occurred very early after stem cell infusion (median of 19 days), and preceding the onset of TMA or aGVHD. Unfortunately, data on cytokine release syndrome (CRS) were not systematically collected, preventing an assessment of its potential contribution to ECE. Notice however that, no ECE were documented between day 0 and +3, and all ECE observed during the study conduction occurred after PTCY administration. However, given the inflammatory nature of CRS, this represents a relevant limitation, as some cardiac manifestations may be triggered or exacerbated by post-transplant inflammatory responses.

The univariate analysis exploring risk factors for ECE further revealed that patients with pre-existing cardiovascular risk factors, such as HTN and DLP, were at a higher risk of developing ECE. However, these factors did not retain statistical significance in the MVA. Interestingly, a history of prior cardiac disease did not significantly correlate with ECE occurrence. These findings emphasize the need for proactive measures to address sedentary behavior and aggressively control cardiovascular risk factors prior to transplantation (8, 11, 25). Moreover, the lack of differences on ECE occurring in patients with and without cardiac morbidity highlights the importance of conducting pre-transplant assessments by Cardiology departments in high-risk patients to implement specific monitoring if needed.

Another finding of interest was the significantly lower rate of CMV reactivation in patients who developed ECE. Although this association reached statistical significance, its clinical relevance is unclear. One potential explanation could be the more widespread use of letermovir prophylaxis in recent years, possibly more frequently administered to patients in the ECE group. However, as letermovir use was not systematically recorded in the dataset, we were unable to evaluate its potential influence on this association. Therefore, we consider this finding to be an incidental observation rather than one directly linked to cardiotoxicity mechanisms. Further studies with detailed data on antiviral prophylaxis would be required to clarify this relationship.

Despite the low incidence of ECE, these complications had a significant impact on NRM and a trend toward lower OS. Nevertheless, although cardiac complications were observed, the cohort exhibited favorable OS and a low incidence of severe aGVHD (10.6% for grade 3-4), highlighting the efficacy of PTCy as a GVHD prophylactic strategy in haplo-HCT. While our findings provide important insights into the incidence and impact of ECE in haplo-HCT, the lack of standardized post-transplant cardiac monitoring may have influenced the detection of subclinical events. Without routine biomarker assessments or echocardiographic follow-ups, some asymptomatic cases could have gone unnoticed, potentially underestimating the true burden of cardiac toxicity. This highlights the need for future studies to incorporate structured cardiac surveillance strategies, which could refine risk assessment and improve early intervention in this population.

The results of this study are important as they highlight the need for robust post-transplant cardiac monitoring protocols, including the measurement of cardiac biomarkers and post-transplant echocardiograms, particularly for patients with pre-existing cardiovascular risk factors. These measures are crucial for early detection of asymptomatic cardiac toxicity and could help reduce morbidity and mortality in haplo-HCT recipients. However, there are notable limitations in this analysis that should be acknowledged.

The retrospective design and the relatively small number of ECE cases (n=23) are key limitations. Additionally, the absence of cardiac biomarker measurements and post-transplant echocardiography may have hindered our ability to detect subclinical cardiac events, such as silent ischemia or minor myocardial injury, which could lead to an underestimation of the true incidence and impact of ECE. Moreover, the lack of data on cumulative anthracycline doses prevents an evaluation of their potential role in ECE development. Considering the negative impact of ECE on outcomes, future studies incorporating detailed chemotherapy exposure data, along with systematic cardiac surveillance and CRS assessments, may provide further insight into the contribution of anthracyclines to the development of ECE.

Although ECEs are infrequent, they significantly impact NRM and OS, emphasizing the importance of prevention and early management. The integration of cardiac monitoring strategies, along with strict management of cardiovascular risk factors, could improve clinical outcomes in haplo-HCT recipients. With the universal adoption of PTCY and the favorable survival rates observed in this cohort, efforts to mitigate cardiac complications should remain a priority in clinical practice and future research.

## Data Availability

The original contributions presented in the study are included in the article/supplementary material. Further inquiries can be directed to the corresponding author.

## In memoriam

We dedicate this work to Dr. Alberto López-García, an exceptional hematologist whose dedication to cardiac toxicity research and patient care continues to inspire us.

## References

[1] Braverman AC Antin JH Plappert MT Cook EF Lee RT. Cyclophosphamide cardiotoxicity in bone marrow transplantation: a prospective evaluation of new dosing regimens. J Clin Oncol. (1991) 9:1215–23. doi: 10.1200/JCO.1991.9.7.1215 2045862

[2] Ishida S Doki N Shingai N Yoshioka K Kakihana K Sakamaki H. The clinical features of fatal cyclophosphamide-induced cardiotoxicity in a conditioning regimen for allogeneic hematopoietic stem cell transplantation (allo-HSCT). Ann Hematol. (2016) 95:1145–50. doi: 10.1007/s00277-016-2654-6 27079957

[3] Marumo A Omori I Tara S Otsuka Y Konuma R Adachi H. Cyclophosphamide-induced cardiotoxicity at conditioning for allogeneic hematopoietic stem cell transplantation would occur among the patients treated with 120 mg/kg or less. Asia Pac J Clin Oncol. (2022) 18:e507–e14. doi: 10.1111/ajco.13674 35289086

[4] Rotz SJ Collier P Hamilton BK. Post-transplantation cyclophosphamide: an old nemesis to a new transplant paradigm? JACC CardioOncol. (2021) 3:260–2. doi: 10.1016/j.jaccao.2021.04.004 PMC8352273 34396332

[5] Dulery R Mohty R Labopin M Sestili S Malard F Brissot E. Early cardiac toxicity associated with post-transplant cyclophosphamide in allogeneic stem cell transplantation. JACC CardioOncol. (2021) 3:250–9. doi: 10.1016/j.jaccao.2021.02.011 PMC8352028 34396331

[6] Yeh J Whited L Saliba RM Rondon G Banchs J Shpall E. Cardiac toxicity after matched allogeneic hematopoietic cell transplant in the posttransplant cyclophosphamide era. Blood Adv. (2021) 5:5599–607. doi: 10.1182/bloodadvances.2021004846 PMC8714723 34592759

[7] Perez-Valencia AI Cascos E Carbonell-Ordeig S Charry P Gomez-Hernando M Rodriguez-Lobato LG. Incidence, risk factors, and impact of early cardiac toxicity after allogeneic hematopoietic cell transplant. Blood Adv. (2023) 7:2018–31. doi: 10.1182/bloodadvances.2022008792 PMC10188633 36453637

[8] Lin CJ Vader JM Slade M DiPersio JF Westervelt P Romee R. Cardiomyopathy in patients after posttransplant cyclophosphamide-based hematopoietic cell transplantation. Cancer. (2017) 123:1800–9. doi: 10.1002/cncr.30534 28262921

[9] Salas MQ Cascos E Lopez-Garcia A Perez E Baile-Gonzalez M Martin Rodriguez C. Cardiac events after allo-HCT in patients with acute myeloid leukemia. Blood Adv. (2024) 8:5497–509. doi: 10.1182/bloodadvances.2024013535 PMC11538614 39178345

[10] Salas MQ Cascos E Lopez-Garcia A Perez-Lopez E Baile-Gonzalez M Lopez-Corral L. Cardiac events occurring after allogeneic hematopoietic cell transplantation with post-transplant cyclophosphamide. Study conducted on behalf of the GETH-TC. Bone Marrow Transplant. (2024) 59:1694–703. doi: 10.1038/s41409-024-02414-z 39277653

[11] Kanate AS Majhail N DeFilipp Z Dhakal B Dholaria B Hamilton B. Updated indications for immune effector cell therapy: 2023 guidelines from the american society for transplantation and cellular therapy. Transplant Cell Ther. (2023) 29:594–7. doi: 10.1016/j.jtct.2023.07.002 37422194

[12] Dohner H Wei AH Appelbaum FR Craddock C DiNardo CD Dombret H. Diagnosis and management of AML in adults: 2022 recommendations from an international expert panel on behalf of the ELN. Blood. (2022) 140:1345–77. doi: 10.1182/blood.2022016867 35797463

[13] Lyon AR Lopez-Fernandez T Couch LS Asteggiano R Aznar MC Bergler-Klein J. 2022 ESC Guidelines on cardio-oncology developed in collaboration with the European Hematology Association (EHA), the European Society for Therapeutic Radiology and Oncology (ESTRO) and the International Cardio-Oncology Society (IC-OS). Eur Heart J Cardiovasc Imaging. (2022) 23:e333–465. doi: 10.1093/ehjci/jeac106 36017575

[14] Schoemans HM Lee SJ Ferrara JL Wolff D Levine JE Schultz KR. EBMT-NIH-CIBMTR Task Force position statement on standardized terminology & guidance for graft-versus-host disease assessment. Bone Marrow Transplant. (2018) 53:1401–15. doi: 10.1038/s41409-018-0204-7 PMC6786777 29872128

[15] Harris AC Young R Devine S Hogan WJ Ayuk F Bunworasate U. International, multicenter standardization of acute graft-versus-host disease clinical data collection: A report from the mount sinai acute GVHD international consortium. Biol Blood Marrow Transplant. (2016) 22:4–10. doi: 10.1016/j.bbmt.2015.09.001 26386318 PMC4706482

[16] Jagasia MH Greinix HT Arora M Williams KM Wolff D Cowen EW. National institutes of health consensus development project on criteria for clinical trials in chronic graft-versus-host disease: I. The 2014 diagnosis and staging working group report. Biol Blood Marrow Transplant. (2015) 21:389–401 e1. doi: 10.1016/j.bbmt.2014.12.001 25529383 PMC4329079

[17] Kanda Y. Investigation of the freely available easy-to-use software ‘EZR’ for medical statistics. Bone Marrow Transplant. (2013) 48:452–8. doi: 10.1038/bmt.2012.244 PMC3590441 23208313

[18] Luznik L O’Donnell PV Symons HJ Chen AR Leffell MS Zahurak M. HLA-haploidentical bone marrow transplantation for hematologic Malignancies using nonmyeloablative conditioning and high-dose, posttransplantation cyclophosphamide. Biol Blood Marrow Transplant. (2008) 14:641–50. doi: 10.1016/j.bbmt.2008.03.005 PMC2633246 18489989

[19] Penack O Marchetti M Aljurf M Arat M Bonifazi F Duarte RF. Prophylaxis and management of graft-versus-host disease after stem-cell transplantation for haematological Malignancies: updated consensus recommendations of the European Society for Blood and Marrow Transplantation. Lancet Haematol. (2024) 11:e147–e59. doi: 10.1016/S2352-3026(23)00342-3 38184001

[20] Iqubal A Iqubal MK Sharma S Ansari MA Najmi AK Ali SM. Molecular mechanism involved in cyclophosphamide-induced cardiotoxicity: Old drug with a new vision. Life Sci. (2019) 218:112–31. doi: 10.1016/j.lfs.2018.12.018 30552952

[21] Nishikawa T Miyahara E Kurauchi K Watanabe E Ikawa K Asaba K. Mechanisms of fatal cardiotoxicity following high-dose cyclophosphamide therapy and a method for its prevention. PloS One. (2015) 10:e0131394. doi: 10.1371/journal.pone.0131394 26114497 PMC4482695

[22] Tolosa-Ridao C Cascos E Rodriguez-Lobato LG Pedraza A Suarez-Lledo M Charry P. EASIX and cardiac adverse events after allogeneic hematopoietic cell transplantation. Bone Marrow Transplant. (2024) 59(7):974–82. doi: 10.1038/s41409-024-02270-x 38521885

[23] Martinez-Sanchez J Pascual-Diaz R Palomo M Moreno-Castano AB Ventosa H Salas MQ. Mafosfamide, a cyclophosphamide analog, causes a proinflammatory response and increased permeability on endothelial cells *in vitro* . Bone Marrow Transplant. (2023) 58:407–13. doi: 10.1038/s41409-023-01912-w 36639572

[24] Knapp M Tu X Wu R. Vascular endothelial dysfunction, a major mediator in diabetic cardiomyopathy. Acta Pharmacol Sin. (2019) 40:1–8. doi: 10.1038/s41401-018-0042-6 29867137 PMC6318313

[25] Palomo M Diaz-Ricart M Carbo C Rovira M Fernandez-Aviles F Martine C. Endothelial dysfunction after hematopoietic stem cell transplantation: role of the conditioning regimen and the type of transplantation. Biol Blood Marrow Transplant. (2010) 16:985–93. doi: 10.1016/j.bbmt.2010.02.008 20167280

